# Electron Scattering from Methyl Formate (HCOOCH_3_): A Joint Theoretical and Experimental Study

**DOI:** 10.1021/acs.jpca.3c04636

**Published:** 2023-08-29

**Authors:** Natalia Tańska, Edvaldo Bandeira, Alessandra Souza Barbosa, Kuba Wójcik, Sylwia Dylnicka, Elżbieta Ptasińska-Denga, Czesław Szmytkowski, Márcio H. F. Bettega, Paweł Możejko

**Affiliations:** † Institute of Physics and Applied Computer Science, Faculty of Applied Physics and Mathematics, Gdańsk University of Technology, ul. Gabriela Narutowicza 11/12, 80-233 Gdańsk, Poland; ‡ Departamento de Física, Universidade Federal do Paraná, Caixa Postal 19044, 81531-980 Curitiba, Paraná, Brazil

## Abstract

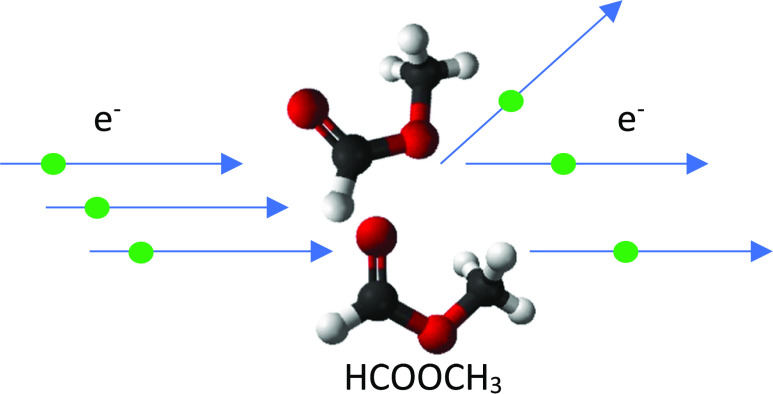

Elastic low-energy electron collisions with methyl formate have been studied theoretically at the level of various theories. The elastic integral cross section was calculated using Schwinger multichannel and R-matrix methods, in the static-exchange and static-exchange plus polarization levels of approximations for energies up to 15 eV. The absolute total cross section for electron scattering from methyl formate has been measured in a wide energy range (0.2–300 eV) using a 127° electron spectrometer working in the linear transmission configuration. The integral elastic and the absolute total cross sections display a π* shape resonance at around 1.70–1.84 eV, which can be related to the resonance visible for formic acid, and a broad structure located at 7–8 eV, which can be associated to a superposition of σ* shape resonances. Our results were compared with theoretical and experimental results available in the literature and with the results of electron collisions with formic acid. The additivity rule was used to estimate the total cross section of methyl formate and the results agree well with the experimental data.

## 1 Introduction

Methyl formate (HCOOCH_3_) is widely used in the synthesis of molecules like formic acid,^1^ acetic acid,^2^ formamides,^3^ and their derivatives. It is also investigated as a surrogate of biodiesel, in particular in the studies of the combustion mechanism.^4^ From the fundamental point of view, it is the simplest ester, a methylated derivative of formic acid, and an isomeric form of acetic acid and glycolaldehyde, which make it an interesting benchmark for the properties of other simple organic molecules.

Methyl formate has been detected toward many interstellar sources, including hot, and prestellar cores,^5,6^ where the prevailing conditions cause the formation of icy grains composed of simple chemical compounds.

Attention has been brought to the unusual differences in the amount and angular distribution of the three mentioned isomers, HCOOCH_3_, CH_3_COOH, and HOCH_2_CHO, in the hot molecular core Sgr B2(N)-LMH. It was found that methyl formate is the most abundant isomer in LMN in the ratio of 1864:103:1 (HCOOCH_3_/CH_3_COOH/HOCH_2_CHO), whereas the source of glycolaldehyde is the most diffuse, extended to 60” in diameter.^8,9^ Methyl formate has also been found in comets,^7^ which are of particular interest due to their possible role in chemical evolution on Earth, recently revived in the light of the discovery of glycine in the coma of the 67P comet.^11^

Interstellar ices, processed by cosmic radiation, are thought to be molecular factories through the reactions of basic compounds like H_2_O, NH_3_, or CO_2_.^12^ Low-energy electrons, produced among secondary species in large amounts^13^ due to the interaction of radiation and matter, are thought to play an important role in inducing chemical reactions occurring in interstellar ices. Their efficiency differs from that of the reactions driven by UV radiation, due to the nonresonant character of excitations, as well as more open reaction channels. As for the latter, one should mention in particular singlet–triplet transitions and dissociative electron attachment (DEA), a process unique for electron-molecule interactions. Methyl formate has been detected in many experiments simulating cosmic conditions, in which ice mixtures or pure condensed methanol-imitated interstellar icy grains were bombarded with high-energy radiation involving protons^15^ and heavy ions,^14^ but also low-energy electrons.^16^

Electron collisions with HCOOCH_3_ in gas phase have been studied both experimentally and theoretically. de Souza et al.^17^ reported elastic cross sections in the 30–1000 eV energy range determined with the relative-flow technique, and elastic and inelastic cross sections obtained with the molecular complex optical potential (MCOP) method combined with Pade approximation, for 1–500 eV energy range. Feketeová et al.^19^ investigated the DEA to methyl formate with a high-resolution electron monochromator and a quadrupole mass spectrometer. In the work of Ragesh Kumar and co-workers,^18^ cross sections for DEA to HCOOCH_3_ were reported and electron energy loss spectra measured with an electrostatic spectrometer were used for obtaining the elastic and vibrationally inelastic cross sections, and further, the π* resonance was characterized with the complex absorbing potential approach combined with multistate multireference perturbation theory. To our knowledge, the only study of total cross section is that of de Souza et al.^17^ and no experimental data are available.

In this joint theoretical and experimental work, we calculated elastic cross sections using the Schwinger multichannel and R-matrix methods, which are two *ab initio* methods well established in the literature. The cross sections were computed at the static-exchange and static-exchange plus polarization approximations, for energies up to 15 eV. We also measured total absolute cross sections for energies ranging from 0.2 up to 300 eV. In particular, our results present a π*-shape resonance located at around 2 eV and belonging to the *A*″ symmetry of the *C*_s_ group. The present results were compared with previous results from de Souza et al.^17^ As methyl formate is a methylated derivative of formic acid, we also compared the present results with elastic and total cross sections of formic acid (HCOOH), and discussed the effect of methylation on the cross sections of methyl formate.

The remainder of this manuscript is as follows: In the next section, we present the theoretical formulation of the R-matrix and the Schwinger multichannel methods, the computational procedures and models employed in the calculations, and the experimental procedures. In the following section, we present the calculated elastic integral and differential cross sections and the absolute total cross section measurements. We close the paper with a brief summary of our findings.

## 2 Theoretical Calculations

### 2.1 R-Matrix Method

In the R-matrix method,^20,28^ it is assumed that a molecule’s electron density can be contained inside a sphere with a finite radius. Once this radius is determined, solving the scattering problem can be divided into two stages: (1) consideration of the system of *N* + 1 indistinguishable electrons inside the sphere (*N* – the number of target electrons), and (2) outer region calculations, in which the scattered electron simply interacts with the static potential. Below, we dive a bit more into details for each of the steps.

### 2.2 Inner Region

In the inner region, that is inside the sphere of radius *a*, the main goal is to find the **R**-matrix basis functions ψ_*k*_ and poles *E*_*k*_, which form eigenpairs of the  operator.  is the Hamiltonian of the whole system in the fixed-nuclei approximation
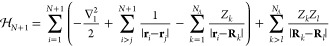
1where **r** and **R** are the coordinates of electrons and nuclei, respectively, *Z* is the atomic number, and *N*_A_ is the number of atoms that the molecule consists of. Bloch operator  is added to ensure hermicity in the inner region; see e.g., ref (20). In UKRmol+ suite implementation,^10^ the basis functions ψ_*k*_ take the following form
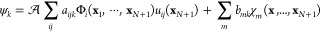
2where Φ_*i*_ are the target states, *u*_*ij*_ are the discretized continuum orbitals, and the second summation goes over the *L*^2^ integrable functions, constructed from target molecular orbitals (occupied and virtual) only. *L*^2^ terms contribute the most to the description of the resonant states. For the continuum description, Gaussian-type orbitals (GTOs) were used in this work. The radial part of the center of mass-centered GTOs for each momentum number *l* consists of the set of Gaussian functions, fitted to the particular Bessel function.^29^ The *ij* subscript is added to emphasize that the inclusion of the continuum orbital of a given symmetry depends on the symmetry of the target state. Finally,  is the antisymmetrization operator and the coefficients *c*_*ijk*_ and *b*_*mk*_ are determined through the diagonalization of the  operator. Basis functions ψ_*k*_ are then used to construct boundary amplitudes *f*, defined as the projection of the *k*-th basis function on the *p*-th scattering channel^10^
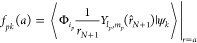
3where Φ_*i*_p__ are the target states from eq 2, now associated with a particular channel *p*, *r*_*N* + 1_ and **r̂**_*N* + 1_ are the radial and angle coordinates of the scattered electron, respectively, and *Y*_*l,m*_ is the real spherical harmonic. Boundary amplitudes can be subsequently inserted into the expression for the **R**-matrix^20^
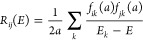
4

For the *L*^2^ functions two types of configurations were used
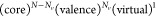
5
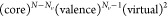
6

The first type is used for static-exchange (SE) approximation, and adding the second one provides inclusion of polarization in the model (SEP). The total scattering wavefunction Ψ can be obtained as a linear sum of basis functions from eq 2.^10^

### Outer Region

In the outer region, the total wavefunction reduces to^10^
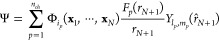
7where *F*_*p*_ is the reduced radial function of the scattered electron in the outer region. In the Hamiltonian  we can separate terms describing the scattered electron, and after some standard operations^20,28^ we obtain a system of differential equations for the reduced radial functions *F*_*p*_(*r*) coupled with the *p*-th scattering channel^20^
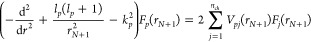
8where *k*_*p*_^2^ = 2(*E* – *E*_*p*_); *E* and *E*_*p*_ are the scattering and channel energy, respectively. For *V*_*pj*_ potential, multipole expansion can be applied, whose coefficients depend on the target multipole moments and their formulas were introduced in ref (20). The procedure for obtaining the **K**-matrices (and other scattering quantities) is to propagate^21^ the **R**-matrix obtained for the R-matrix radius *r* = *a* (in the last step of the inner region calculations) and use it as a boundary condition for the asymptotic expansion of the solution to eq 8, the exact form of which can be found in ref.^30^ From the **K**-matrix, one can calculate the **S**-matrix, **T**-matrix, and the corresponding total cross section (calculated as the sum over all transitions from singlet ground state)
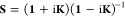
9
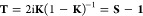
10
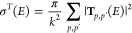
11where the summation goes over open channels *p* and *p*′. It should be noted that most of the steps described above are performed for each irreducible representation of the molecule’s symmetry point group separately. Another important quantity, directly related to the **S**-matrix, is the time-delay (**Q**) matrix
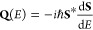
12**Q**-matrix is extremely useful for detecting and analyzing resonant states. Resonances appear as Lorentzian peaks in the eigenvalues of **Q**-matrix, *q*, as a function of energy
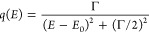
13where *E*_0_ and Γ are the position and width of a resonance, respectively. Fitting the appropriate Lorentzian function provides the resonance parameters. For calculations of **Q**-matrix, its eigenvalues and eigenvectors, as well as function fitting in the vicinity of the resonance, the TIMEDELn program was used.^27^

### 2.3 Calculation Details

Target orbitals were obtained with the Hartree–Fock method in a 6-311G** basis set, using the MOLPRO program.^22−24^ Calculations were performed at the geometry optimized in MP2/cc-pVTZ taken from ref (31). Experimental geometry^31^ and other basis sets (cc-pVDZ and diffuse ones) were also tested. The final computational setup was chosen due to good agreement with the experiment without making the calculations too large. The obtained valence electronic structure of the ground state is (5–10*a*′)^12^ (1*a*″)^2^ (11*a*′)^2^ (2*a*″)^2^ (12*a*′)^2^ (3*a*″)^2^ (13*a*′)^2^, in accordance with Nunes et al.^32^ 37 unoccupied orbitals were retained in the calculations and all single transitions from the valence to virtual orbitals were included. The R-matrix radius was set to 18a_0_, although other radii were also tested, giving very similar results. For the continuum basis, we used Gaussian exponents optimized by Tarana et al.^25^ (for *l* < 5) and by Loupas et al.^26^ (for *l* = 5). The radius at which asymptotic expansion was applied was set to 100*a*_0_. Calculations were performed in quadruple precision. Methyl formate is a polar molecule, having a dipole moment of 1.77 D determined experimentally,^31^ compared to 1.90 D in the current HF calculations. Therefore, Born correction for the rotating dipole was added as the difference between the total analytic cross section and cross section obtained for partial waves of *l* ≤ 5.^33^

### 2.4 Schwinger Multichannel Method

#### 2.4.1 Theory

The Schwinger multichannel (SMC) method^34,35^ and its current implementations^36,37^ have been recently reviewed and here we will only describe the most relevant aspects of the method for the present calculations. The SMC method is a variational approximation for the scattering amplitude. The resulting expression for the scattering amplitude in the body frame of the target is
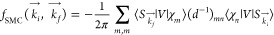
14where |*S*_*k⃗*_*i*(*f*)__⟩, an eigenstate of the the unperturbed Hamiltonian *H*_0_, is given by the product of a target state and a plane wave with momentum *k⃗*_*i*(*f*)_, and {|χ_*m*_⟩} is the basis set composed of (*N* + 1)-electron symmetry-adapted Slater determinants constructed from the product of target states with single-particle functions, also known as configuration state functions (CSFs). The *d*_*mn*_ matrix elements are given by
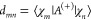
15and the *A*^(+)^ operator is given by
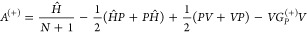
16where *Ĥ* ≡ *E* – *H* is the difference between the total collision energy and the full Hamiltonian of the system with *H* = *H*_0_ + *V*, *P* is a projection operator onto the open-channel space, *V* is the interaction potential between the incident electron and the target, and *G*_*P*_^(+)^ is the free-particle Green’s function projected on the *P* space. For elastic scattering we consider only the target ground-state channel as open. In this case, *P* = |Φ_1_⟩⟨Φ_1_|, where |Φ_1_⟩ is the target ground state described at the Hartree–Fock level.

The SMC calculations are presented in the static-exchange (SE) and in the static-exchange plus polarization (SEP) approximations. In the SE approximation, the CSFs are constructed as
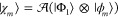
17where |ϕ_*m*_⟩ is a scattering orbital represented by an unoccupied molecular orbital and  is the antisymmetrization operator of (*N* + 1) electrons. In the SEP approximation, the active space is augmented by CSFs constructed as
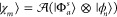
18where |Φ_*a*_^*s*^⟩ (*a* ≥ 1) is a virtual single excitation of the target, obtained by the excitation of one electron from a valence-occupied (hole) orbital to an unoccupied (particle) orbital, with spin coupling *s* (*s* = 0 for singlets or *s* = 1 for triplet), and |ϕ_*n*_⟩ is also a scattering orbital.

The Cartesian–Gaussian-type functions employed were used as the single-particle basis in the SMC, as *L*^2^ functions, and, as a consequence, the long-range dipole potential is truncated. In order to circumvent this issue and enhance the accuracy of the calculated cross sections, a Born-closure procedure^37^ is employed to describe the higher partial waves. In the Born-closure procedure the low partial waves are described by the SMC method up to a certain *l*_SMC_ value, while the higher partial waves are included in the calculations through the scattering amplitude of the dipole potential computed in the first Born approximation (FBA) from *l*_SMC_ + 1 to ∞.

#### 2.4.2 Computational Details

The geometry of the molecular ground state was optimized in the *C*_s_ point group at the second-order Møller–Plesset perturbation theory level with the aug-cc-pVDZ basis set using the package GAMESS.^38^ The norm-conserving pseudopotentials of Bachelet, Hamann, and Schlüter^39^ were used to replace the core electrons of the carbon and the oxygen atoms. The uncontracted Cartesian–Gaussian functions used for the carbon and oxygen atoms contain 5*s*5*p*3*d* functions and were published elsewhere.^40^ For the hydrogen atoms, we employed the 4s/3s basis set of Dunning Jr.^41^ with one additional *p*-type function with exponent 0.75. Additionally, we included additional Cartesian–Gaussian functions in three extra chargeless centers^42^ placed along the C=O, C–H, and O–C bonds, with exponent values of 0.100, 0.0250, and 0.00625 for the *s*-type functions, 0.0500 and 0.0125 for the *p*-type functions, and 0.0250 for a *d*-type function.

The canonical Hartree–Fock orbitals were employed as scattering orbitals in the SE approximation, while the modified virtual orbitals (MVOs)^43^ generated from the diagonalization of a cationic Fock operator with charge +4 were employed to represent the particle and the scattering orbitals in the SEP approximation. To build the CSFs employed in the SEP calculations, we included all singlet and triplet excitations arising from the 12 valence-occupied (hole) orbitals to the lowest 53 MVOs, employed as particle orbitals. The same set of MVOs were employed as scattering orbitals, resulting in 17 677 CSFs for the *A*′ symmetry. For the resonant *A*″ symmetry, we included all single excitations by preserving the spatial and spin symmetry of the ground state, and only one orbital representing the π* resonant orbital was employed as the scattering orbital, resulting in 1576 CSFs for this symmetry. Thus, a total 19 253 CSFs were employed in the SMC-SEP calculation.

The calculated value of the permanent dipole moment is 2.01 D, which is higher than the experimental value of 1.77 D.^31^ As mentioned above, to include properly the effects of the dipole moment potential in our calculations, the partial waves up to a certain *l*_SMC_ value are obtained from the SMC calculations, while higher partial waves are obtained from the scattering amplitude of the dipole potential computed in the first Born approximation. The value of *l*_SMC_ depends on the incident electron energy, and in the present calculations the following values were employed: *l*_SMC_ = 1 for impact energies up to 0.90 eV, *l*_SMC_ = 3 from 1.00 to 2.02 eV, *l*_SMC_ = 4 from 2.03 to 4.00 eV, *l*_SMC_ = 5 from 4.50 to 5.50 eV, *l*_SMC_ = 6 from 6.00 to 8.00 eV, and *l*_SMC_ = 7 from 8.50 to 15.00 eV.

## 3 Experimental Procedure and Uncertainty Analysis

### 3.1 Experimental Procedure

The total cross sections for electron scattering from the methyl formate, (HCOOCH_3_), molecules presented here have been obtained using a cylindrical electron spectrometer with the linear electron-transmission method under single-collision conditions. The used apparatus and the measurement procedures used in the present experiment have been described in detail in our previous works^44−46^ and only a brief outline will be provided here.

A tunable-energy monoenergetic (Δ*E* ∼ 80 meV) electron beam produced with a thermionic gun and formed in a system of electrostatic lenses coupled to an energy-dispersing 127° electrostatic deflector was directed into a scattering cell, where its intensity was attenuated by the presence of the vapor sample under investigation. Those electrons that leave the cell through the exit aperture in the forward direction are energy discriminated by the retarding-field filter and eventually detected with the Faraday cup. The acceptance angle of the employed electron detector system as seen from the center of the scattering cell, which is defined by the lens aperture, is near 0.8 msr. The absolute total cross section (TCS), *Q*(*E*), for the scattering of electrons of a given energy *E* from the target molecules, is determined from the attenuation of the transmitted beam intensity through the Bouguer–de Beer–Lambert (BBL) relationship
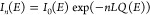
19

where *I*_*n*_(*E*) and *I*_0_(*E*) are the intensities of the electron beam transmitted across the scattering cell measured with and without the target in the cell, respectively. *L* = 30.5 mm is the path length of electrons in the reaction volume and *n* is the absolute number density of the target vapors. The number density, *n*, is determined taking into account the thermal transpiration effect,^47,48^ using the ideal gas formula from the measurements of the gas target pressure, *p*_*t*_, and temperatures of the cell (*T*_c_ = 310–320 K) and the capacitance manometer head (*T*_m_ = 322 K), which finally leads to the following formula for TCS
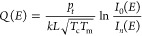
20

The electron spectrometer is housed in a vacuum chamber pumped down to a base pressure of about 40 μPa. The magnetic field along the whole electron trajectory is reduced to below 0.1 μT with the system of Helmholtz coils. To recognize and eliminate multiple electron collisions, the TCS measurements have been carried out at different target-vapor pressures inside the scattering cell. For target pressures in the range from 80 to 200 mPa, no systematic variation of the measured TCSs with pressure is observed; thus, one can assume that multiple scattering events are not significant.

The energy scale has been calibrated against the oscillatory structure at around 2.3 eV in the transmitted current when molecular nitrogen was admixtured to the target under study. The declared inaccuracy of the energy scale (∼0.1 eV) is higher than that resulting directly from the calibration due to the shift in energy, perceptible in the course of the long-lasting experiment.

A commercially supplied (CPAchem) sample of high-purity (≥99.5%) methyl formate was distilled by freeze–pump–thaw repetitive cycles before use to remove volatile impurities. The target vapor was admitted into the spectrometer via a variable leak valve and alternately into the reaction cell; the outer vacuum volume, and thus the pressure in the region of the electron optics, was maintained constant (below 0.6 mPa) whether or not the target was present in the cell, which ensured a stable primary electron-beam intensity during both phases of the intensity measurements. Due to a low vapor pressure of methyl formate at room temperature, the sample handling system was maintained at an elevated temperature of about 315 K.

The final TCS value at each electron-impact energy was derived as the weighted mean of results obtained in independent series (6–14) of individual runs (usually 8–10 in a series). The statistical variations of the measured TCS, estimated as one standard deviation of the weighted mean value from TCS values obtained in different series, do not exceed 1% below 100 eV and gradually increase up to nearly 2% at the highest electron-impact energies applied.

### 3.2 Uncertainty Analysis

The accuracy of the TCS measured with the transmission method is mainly determined by the possible systematical uncertainties.^49^ One of the most important issues is the effusion of the target molecules through the orifices of the reaction cell, which leads to inhomogeneous target density distribution, *n*, along the electron trajectory in the cell, and hence makes it difficult to determine the effective path length, *L*, of electrons across the sample volume. To estimate the uncertainty related to the factor *nL* in the BBL formula, we followed the method adopted from ref (50) to the present experimental conditions. The calculations show that the target pressure drop in the vicinity of the scattering cell orifices is nearly compensated by the elongation of the effective path length. Another possible uncertainty in the electron-transmission experiment relates to the electron-beam intensity measurements and energy scale calibration. The most serious problem is connected to the forward-angle scattering effect, i.e., inability to discriminate against electrons that are scattered elastically through small angles in the forward direction and that contribute to the measured transmitted current, resulting in the lowering of the measured TCS.^51^ The applied retarding-field filter prevents only the electrons scattered inelastically in the forward direction from being detected together with those unscattered. It must be noted here that the reported TCS data are not corrected for the forward-angle scattering effect.

The overall systematical uncertainty in the presented absolute TCS, estimated as the sum of potential systematic errors of all quantities taken in the experiment, amounts to 15% below 1.5 eV, up to 9% between 1.5 and 5 eV, 7% within 5–20 eV, about 6% between 20 and 100 eV, and increases to 8% at higher energies.

## 4 Results and Discussion

### 4.1 Total Cross Section

**Figure 1:**
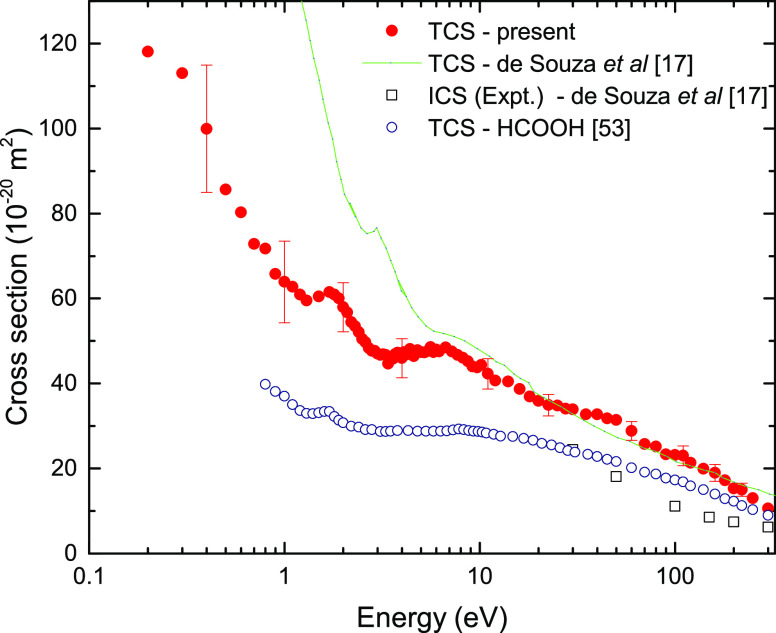
Present experimental TCS for HCOOCH_3_. ICS and TCS for HCOOCH_3_ of de Souza and co-workers^17^ and experimental TCS for HCOOH from ref (53) are also depicted for comparison.

The experimental total cross section (TCS) for electron scattering from methyl formate in the whole investigated energy range (0–300 eV) is depicted in Figure 1 and presented in the numerical form in Table 1. The present experimental total cross section is larger in magnitude than the numerically integrated experimental elastic DCS of de Souza et al.,^17^ as expected. It is, however, in very good agreement above 10 eV with the calculated *grand*-TCS, also presented in their work (see Figure 1 for comparison). A weak shoulder around 40 eV is visible only in our results. Its origin at the moment is rather unclear, but some contribution to that structure may arise due to the increasing cross section for the ionization process, which reaches the maximum at 100 eV of magnitude 7.6 × 10^–20^ m^2^.^52^ To our knowledge, there are no other comparative experimental data concerning electron scattering from HCOOCH_3_.

**Table 1:** Total Cross Section for Electron-HCOOCH_3_ Collisions in 10^–20^ m^2^ Units

energy (eV)	TCS	energy (eV)	TCS	energy (eV)	TCS
0.2	118.0	3.3	46.7	11	42.3
0.3	113.0	3.4	44.6	12	40.7
0.4	99.9	3.5	46.3	14	40.5
0.5	85.7	3.6	45.8	16	38.7
0.6	80.3	3.7	47.0	18	37.0
0.7	72.8	3.8	47.2	20	35.9
0.8	71.7	3.9	46.3	22.5	34.9
0.9	65.7	4.0	46.0	25	34.8
1.0	63.9	4.1	47.4	27.5	34.1
1.1	62.7	4.2	46.8	30	33.9
1.2	60.9	4.4	48.1	35	32.7
1.3	59.5	4.6	46.4	40	32.7
1.5	60.5	4.8	47.8	45	31.8
1.7	61.5	5.0	47.4	50	31.4
1.8	60.9	5.2	47.3	60	28.9
1.9	60.1	5.4	47.6	70	25.7
2.0	57.9	5.6	48.6	80	25.2
2.1	56.7	5.8	47.4	90	23.3
2.2	54.4	6.0	48.0	100	23.2
2.3	53.5	6.2	47.6	110	23.0
2.4	52.1	6.7	48.5	120	21.4
2.5	50.5	7.2	47.5	140	20.0
2.6	49.7	7.7	46.7	160	19.0
2.7	48.5	8.2	46.1	180	17.2
2.8	47.7	8.7	45.3	200	15.3
2.9	48.0	8.7	45.3	220	15.0
3.0	47.0	9.2	44.0	250	13.0
3.1	46.8	9.7	43.7	300	10.6
3.2	46.8	10.2	44.4		

**Figure 2:**
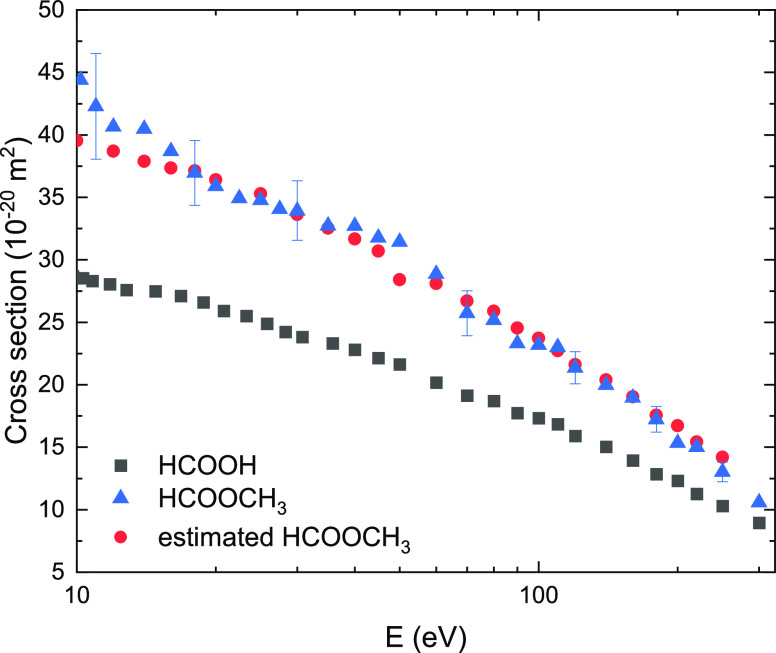
TCS for HCOOCH_3_, estimated with the additivity rule, compared with actual experimental results for HCOOCH_3_ and HCOOH.

As mentioned above, methyl formate (HCOOCH_3_) is a methylated derivative of formic acid; therefore, it is natural to compare the cross sections of these two compounds. The experimental TCS values for methyl formate and formic acid^53,54^ are compared in Figure 1. According to the results, the position of the π* shape resonance does not change after adding a methyl group to HCOOH, while the second broad peak is clearly more pronounced for HCOOCH_3_. At high enough energies, the cross section for HCOOCH_3_ is expected to be larger than for HCOOH simply due to the difference in the geometrical size, and above 20 eV no effective resonant processes should occur. Therefore, we approximated the TCS for HCOOCH_3_ with the additivity rule,^55^ applying the following formula (the analysis for the preliminary results have been reported at the SPIG Conference^56^)
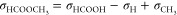
21where σ_HCOOH_ is directly taken from (53), and σ_H_ and σ_CH3_ are estimated as half of the molecular hydrogen^57^ and ethane^58^ cross section, respectively. It is worth noting here that all of these data were obtained in our laboratory. The result, shown in Figure 2, is in very good agreement with the original experimental data for HCOOCH_3_, proving the consistency of our measurements.

### 4.2 Integral Elastic Cross Sections

**Figure 3:**
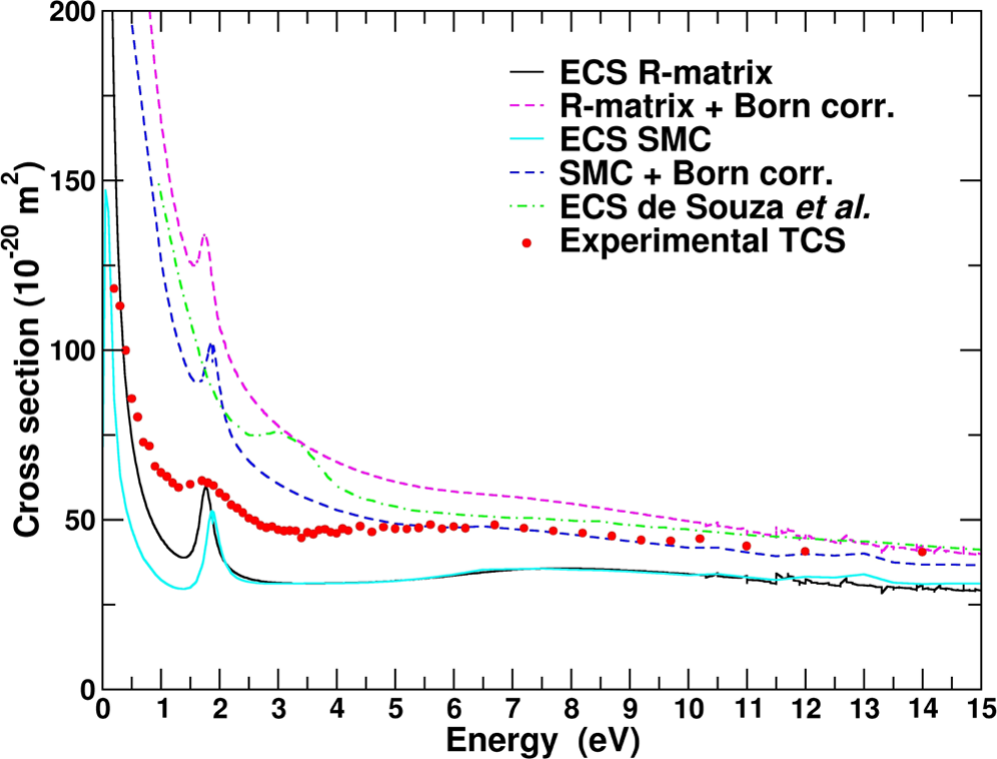
Cross sections for electron scattering on HCOOCH_3_ in the low-energy range: experimental *grand*-total cross section (TCS), elastic cross sections (ECS) obtained with SMC and R-matrix methods with and without Born correction, and ECS obtained with MCOP calculations by de Souza et al.^17^

The elastic integral cross sections obtained with SMC and R-matrix methods in comparison with the experimental TCS for low energies (up to 15 eV) are shown in Figure 3. In general, it is seen that both calculations present an overall good agreement among them and with the experimental TCS. When comparing the Born corrected cross sections for both methods, it is noted that the calculated results differ in magnitude. For energies higher than 3 eV, this is mainly due to the different procedures adopted by both methods to carry out these corrections, since the uncorrected cross sections lie together in this energy range. The origin of the discrepancy is in part due to the different stages at which the correction is applied: in the SMC method the Born-closure procedure is done in the scattering amplitude and in the R-matrix method this procedure is done in the cross section. The use of different values of *l*_max_ in the partial wave expansion of the scattering amplitude and of the cross section in the SMC and R-matrix methods, respectively, in order to proceed with the Born-closure, may also contribute to this discrepancy. At energies below 1 eV, it is noted that both calculated results present a rapid increase as the impact energies go toward zero. It may be noted that this is typical for molecules with a permanent dipole moment. Partial wave analysis (performed for R-matrix calculations) showed that transitions of Δ*l* ≠ 0 contributed most to this trend, confirming its origin.

**Figure 4:**
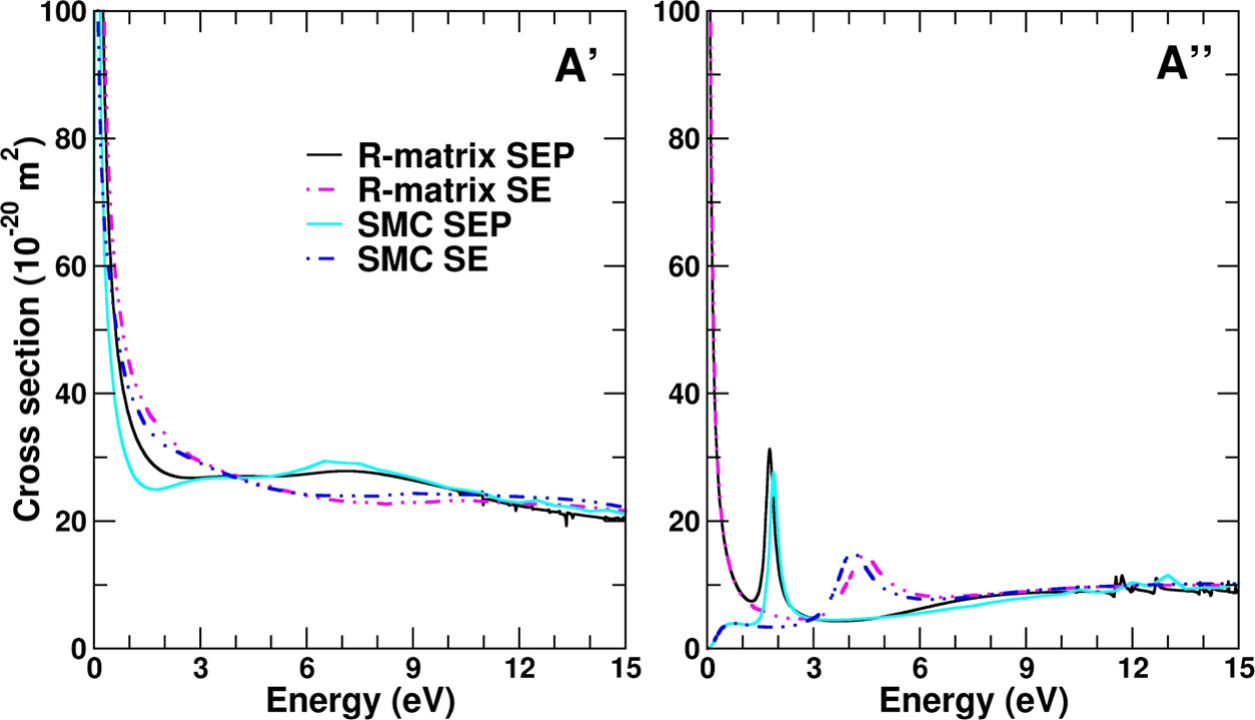
Symmetry decomposition, according to the C_*s*_ symmetry group, of the integral elastic cross sections obtained with SMC and R-matrix methods, in SE and SEP approximations. A′ cross sections are in the left panel, whereas the right panel displays the A″ cross sections. Also shown in the right panel is the resonant π* orbital related to the shape resonance.

The symmetry decomposition of the integral elastic cross sections, shown in Figure 4, reveals that the resonant-like structure, present at around 2 eV, arises from the *A*″ symmetry, whereas the broad structure at around 8 eV is due to the *A*′ symmetry contributions. In the right panel of Figure 4 is also shown the resonant-like orbital related to the π* shape resonance. The difference observed at lower energies, in particular, for the *A*″ symmetry, where the R-matrix cross sections increase as the energy goes toward zero whereas the SMC cross sections decrease, is also very intriguing. This behavior is due to the description of the outer region in the R-matrix calculations. The coupling potential is expressed as a single-center expansion of the Coulomb interaction and usually, terms till the quadrupole moment are retained. If higher-order terms are excluded from the calculations, then R-matrix and SMC calculations lie together even at these lower energies.

Our symmetry-summed theoretical (uncorrected) SEP results are in excellent agreement above 2 eV. Below this energy, the discrepancy, visible in both irreducible representations, arises from the different descriptions of the long-range interaction. Both uncorrected curves lie below the experimental TCS over the whole energy range, which is expected for polar molecules due to the small number of partial waves included in the calculations. Adding the Born correction, however, results in an overestimation of the cross section up to 10 eV. The narrow structures visible above 10 eV in the SEP model most likely arise from incomplete description of the target states (so-called pseudoresonances). The cross sections obtained within both theoretical approaches (i.e., SMC and R-matrix method) in SE approximation are less steep at low energies and also reveal a π* shape resonance, although much wider and higher in energy than at SEP level of theory (4.1 and 4.4 eV in SMC and R-matrix calculations, respectively).

### 4.3 Resonances

**Figure 5:**
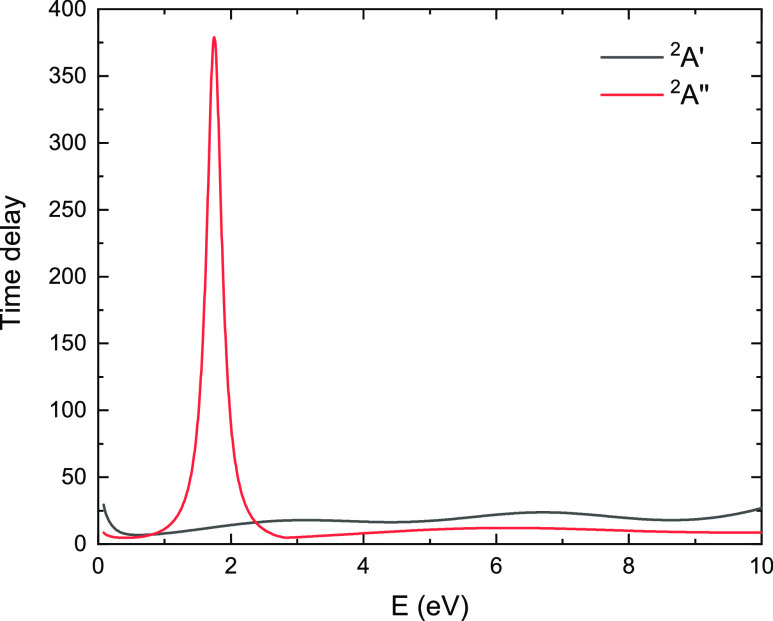
Largest eigenvalue of the time-delay matrix obtained in the SEP approximation in ^2^*A*′ and ^2^*A*″ scattering symmetries.

In Figure 5 we show the energy dependence of the highest eigenvalue of the Q-matrix, obtained in R-matrix calculations, for both irreducible representations. In ^2^*A*″ symmetry, by fitting the Lorentzian function with TIMEDELn program,^27^ resonance at 1.75 eV of 0.30 eV width was detected.

**Figure 6:**
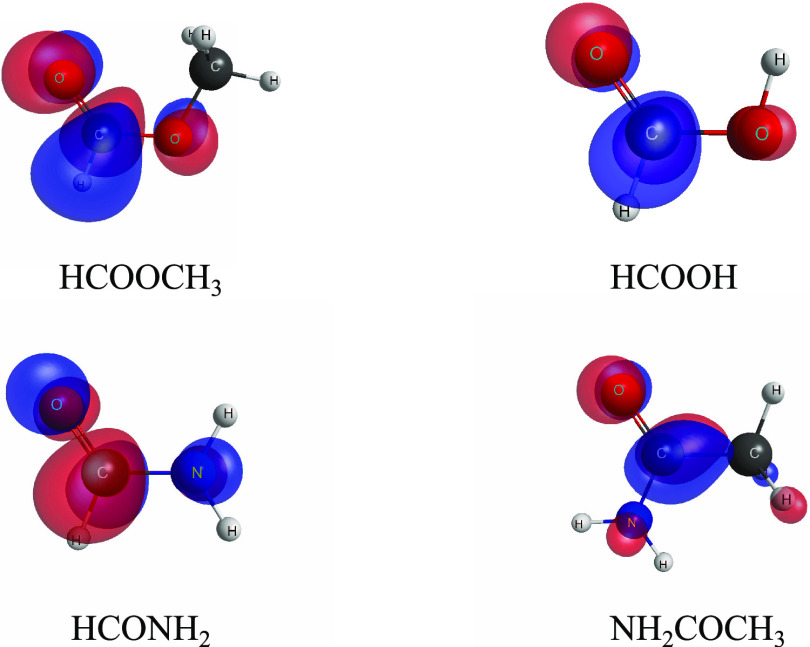
Plot of the π* (LUMO) orbital for methyl formate (HCOOCH_3_), formic acid (HCOOH), formamide (HCONH_2_), and acetamide (NH_2_COCH_3_). The orbitals were generated with MacMolPlt.^62^

The positions of the detected resonant-like structures are summarized in Table 2. For all curves, a prominent peak is observed: around 1.7 eV for TCS and SEP approximations, and around 4 eV for SE models (for the SE results, see Figure 4 and discussion below). This peak corresponds to the shape resonance, which can be approximated as the electron capture to the LUMO π* orbital of ^2^*A*″ symmetry, which is characteristic for species with a carbonyl group and is depicted in Figure 6 for methyl formate, formic acid, formamide, and acetamide. These orbitals were obtained in a Hartree–Fock calculation with optimized geometry at the MP2 level, both calculations with the 6-31G(*d*) basis set using GAMESS.^38^ This structure was also detected in the MCOP calculations of de Souza et al.^17^ at 3 eV, and in the joint experimental and theoretical studies by Ragesh Kumar et al.^18^ at 2.1 and 2.34 eV, respectively. Both mentioned DEA investigations^18,19^ detected a signal for negative ion fragment formation (CH_3_O^–^, CHO_2_^–^, C_2_H_3_O_2_^–^) in the vicinity of this structure, in the 1–4 eV energy range.

**Table 2:** Positions of Resonances (in eV) Detected Experimentally (Exp.) and in SMC and R-Matrix Calculations in SEP Approximation^a^

	present			de Souza et al.^17^	Ragesh Kumar et al.^18^
resonance	exp.	SMC	R-matrix	MCOP	exp.
π*	1.7	1.84	1.75	3.0	2.1
σ*	7.0^b^	7.0^b^	8.0^b^	8.0	

a Positions reported previously in literature are also given for comparison.

b Position of the broad resonant-like feature that may be associated with σ* resonance.

At slightly higher energies, a broad structure centered around 7–8 eV can be observed in both experimental and theoretical results. In the calculated cross sections (R-matrix and SMC within SEP approximation) it has ^2^*A*′ symmetry. This feature may correspond to the overlap of multiple σ* type resonances, which occurs also for other small compounds of biological importance (e.g., furan,^59^ formic acid,^53^ ethane,^60^ propane,^61^ acetone^63,64^). A similar structure was indicated for methyl formate by de Souza et al.,^17^ also at 8 eV. The time-delay analysis for ^2^*A*′ symmetry (Figure 5) shows two relatively weak and broad peaks in the described energy range. The first one was located by TIMEDELn at 3.42 eV, and the second one at 6.71 eV, both having very large widths of 5.44 and 3.94 eV, respectively. The corresponding features were also observed in the time delay obtained for SE approximation (shifted toward higher energies), as well as for different geometry and R-matrix radii (not shown here), but whether these are structures of physical meaning remains unclear. For its precise characterization, a more complex analysis should be used, e.g., an analysis of the poles of the S-matrix (Siegert states).^66^ In the DEA experiment of Feketová and co-workers,^19^ the signal from negative fragments was also measured in the range of 5–14 eV, much stronger than the one at lower energies except for the C_2_H_3_O_2_^–^ fragment. However, since the excitation threshold for HCOOCH_3_ is around 5 eV,^65^ fragments observed in this energy range can also be formed via core-excited states,^19^ which are absent from our calculations.

### 4.4 Differential Cross Sections

**Figure 7:**
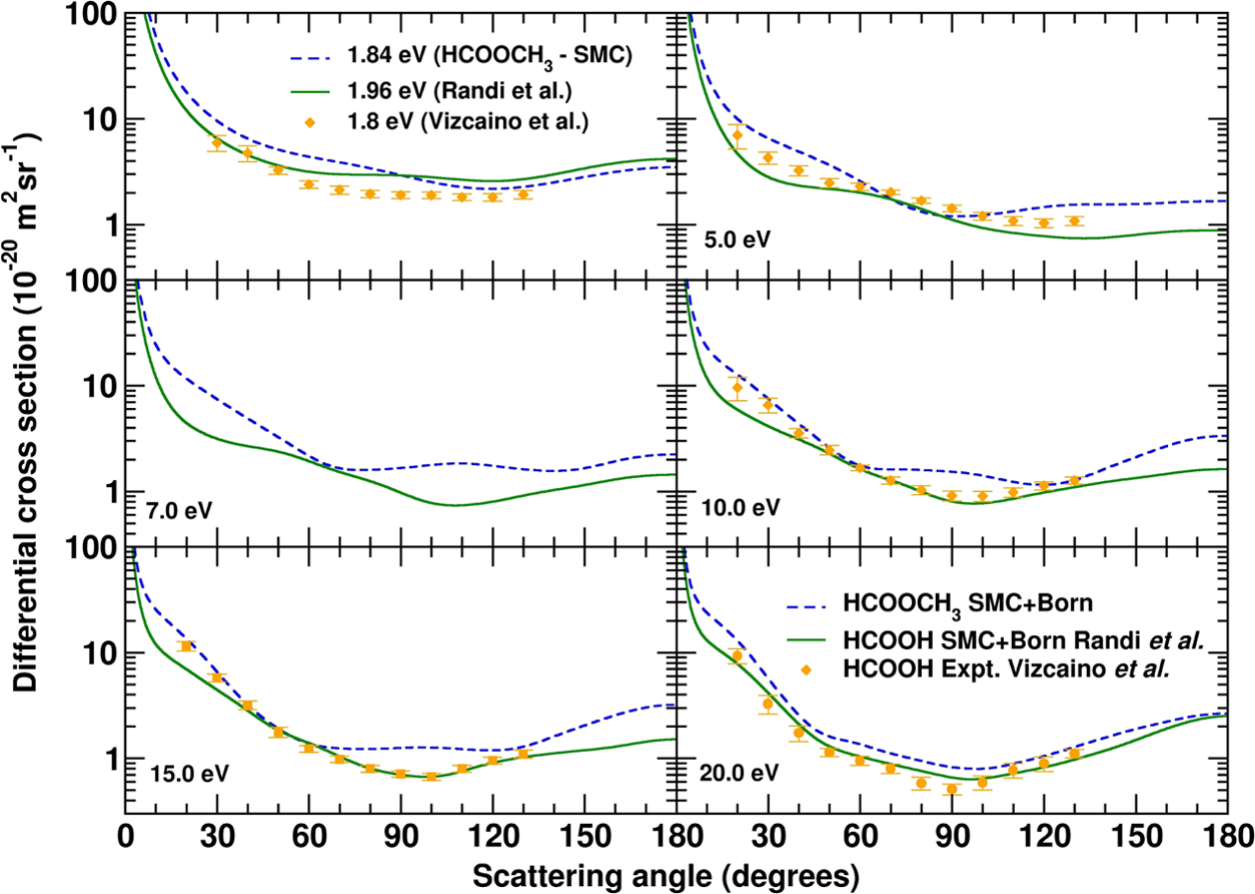
Differential cross sections for electron elastic scattering by methyl formate at selected energies. Also shown are available data for formic acid: theoretical calculations from Randi et al.^67^ and experimental measurements from Vizcaino et al.^68^

In Figure 7 we present our calculated differential cross sections (DCSs) for elastic scattering of electrons by methyl formate at selected energies, as obtained with the SMC method. The results are presented in the SEP + Born approximation, where the long-range effects of the dipole potential are included through the Born-closure procedure. We compare our DCSs with previous calculations for formic acid by Randi et al.^67^ obtained with the SMC method, and with available experimental DCSs for formic acid reported by Vizcaino et al.^68^ In particular, we compare our DCS at 1.84 eV, which corresponds to the energy of the π* shape resonance of methyl formate, with theoretical and experimental DCSs of formic acid also at the energy of the π* shape resonance of formic acid (1.96 and 1.8 eV, respectively). The DCSs agree well in shape and in magnitude at the resonance energy. There are important differences in the magnitude and in shape between the DCSs for methyl formate and formic acid at 5 eV and above, except at 20 eV, where they have a similar shape and differ little in magnitude. The oscillation pattern of the DCSs of methyl formate and formic acid differ in the number of minima at 5, 7, 10, and 15 eV. These differences, in both shape and magnitude, are due to the effect of methylation in methyl formate.

The present DCSs can be used to estimate the possible correction to the TCS due to the forward scattering effect. It is of note that the TCS value with correction can be up to 100 × 10^–20^ m^2^ at 1.8 eV and, at the lower investigated energies, this correction can be even more significant.

## 5 Conclusions

In this joint experimental and theoretical study, we presented the absolute total and elastic integral and differential cross sections for electron scattering by methyl formate. Our calculations employed the SMC and R-matrix methods. Our total and elastic integral cross sections present a π* shape resonance around 1.7–1.84 eV and a superposition of σ* resonances at around 7–8 eV. We estimated the total cross section of methyl formate using the additivity rule, and the results were in very good agreement with the measured cross section. The results obtained with the SMC and R-matrix methods agree well. Methyl formate is a methylated derivative of formic acid, and we also compared the results of these two molecules. In particular, we observed differences in magnitude and in the oscillatory pattern in the differential cross sections, which can be attributed to the effect of methylation. The comparison between the total cross sections of methyl formate and formic acid shows a difference in magnitude, the cross section of methyl formate being bigger due to the molecular size, while the π* resonances are observed at the same energy.
